# The musical environment and auditory plasticity: hearing the pitch of percussion

**DOI:** 10.3389/fpsyg.2013.00768

**Published:** 2013-10-24

**Authors:** Neil M. McLachlan, David J. T. Marco, Sarah J. Wilson

**Affiliations:** Melbourne School of Psychological Sciences, The University of MelbourneMelbourne, VIC, Australia

**Keywords:** inharmonic, pitch, recognition, plasticity, percussion, instrument

## Abstract

Although musical skills clearly improve with training, pitch processing has generally been believed to be biologically determined by the behavior of brain stem neural mechanisms. Two main classes of pitch models have emerged over the last 50 years. Harmonic template models have been used to explain cross-channel integration of frequency information, and waveform periodicity models have been used to explain pitch discrimination that is much finer than the resolution of the auditory nerve. It has been proposed that harmonic templates are learnt from repeated exposure to voice, and so it may also be possible to learn inharmonic templates from repeated exposure to inharmonic music instruments. This study investigated whether pitch-matching accuracy for inharmonic percussion instruments was better in people who have trained on these instruments and could reliably recognize their timbre. We found that adults who had trained with Indonesian gamelan instruments were better at recognizing and pitch-matching gamelan instruments than people with similar levels of music training, but no prior exposure to these instruments. These findings suggest that gamelan musicians were able to use inharmonic templates to support accurate pitch processing for these instruments. We suggest that recognition mechanisms based on spectrotemporal patterns of afferent auditory excitation in the early stages of pitch processing allow rapid priming of the lowest frequency partial of inharmonic timbres, explaining how music training can adapt pitch processing to different musical genres and instruments.

## Introduction

There is a growing body of evidence to suggest that environmental influences can enhance pitch processing abilities. Just as people's ability to discriminate phonemes alters as they learn languages (Liberman et al., 1967), native speakers of tonal languages display enhanced discrimination ability for the pitch contours of words (Stevens et al., 2013). Enhanced pitch perception has also been associated with music training by numerous researchers (Thurlow, 1963). However, these data could not distinguish whether this was due to learning music, or inherited pitch processing abilities that influenced the choice to become a musician. More recently a few studies have reported improvement in pitch matching accuracy with training (Hutchins and Peretz, 2011; McLachlan et al., 2013). In these studies most non-musicians initially displayed a level of pitch matching ability commensurate with the ability to perceive relatively large pitch intervals of around two semitones found in the prosody of European languages (Patel et al., 2006). This is consistent with pitch discrimination ability being defined by the behavioral demands of the environment, and with the more general ability of animals to learn to recognize and discriminate the frequency of sounds that have behavioral significance, such as conditioned reflexes in animals (Ohyama et al., 2003; Weinberger, 2011), and speech perception in humans (Liberman et al., 1967).

However, research on pitch processing mechanisms over the last 50 years has largely assumed that pitch mechanisms behave in a fixed, biologically determined way to all stimuli. Models of pitch processing generated over this period broadly fall into two types; harmonic template matching models, and temporal waveform processing models (de Cheveigné, 2005). Harmonic template matching models of pitch (Goldstein, 1973; Terhardt, 1974; Parncutt, 1989) were proposed to explain the phenomenon of virtual pitch percepts at the fundamental frequency of harmonic complexes when their fundamental is missing. Terhardt (1974) suggested that harmonic templates for pitch processing are learnt from exposure to speech, whereas Shamma and Klein (2000) suggested that they may naturally emerge from non-linearities in the responses of the auditory system to broadband stimuli. However the frequency resolution of harmonic template models is limited by the resolution of the auditory nerve, and autocorrelation models of pitch (Licklider, 1951; Meddis and Hewitt, 1991) were found to produce finer pitch resolution. Furthermore temporal waveform processing models have been supported by observations that brain stem neurobiological mechanisms extract the periodicity of the waveform (Cariani and Delgutte, 1996; Meddis and O'Mard, 2006).

Inharmonic sounds present a particular difficulty for both these classes of pitch models, since their spectra do not fit harmonic templates, and they do not produce stable waveforms with a constant period. Attempts to fit harmonic template models of pitch to inharmonic bell spectra have produced inconsistent results (Terhardt et al., 1982a; Parncutt, 1989; McLachlan et al., 2003), leading to the general belief that inharmonic sounds cannot produce consistent pitch percepts, despite the widespread use of inharmonic tuned percussion instruments in music traditions around the world. Experimental research on pitch processing of inharmonic complexes has been limited to investigating the influence of small changes in frequency of specific partials on pitch perception by a few highly trained participants (Schouten et al., 1962; Ritsma, 1967; Schouten and 't Hart, 1984; Moore and Moore, 2003). It has not explicitly tested whether consistent pitch responses for inharmonic sounds with similar timbres over a range of frequencies (such as in tuned percussion instruments) could be reliably learnt by musicians. Evidence for this would support the proposition that pitch involves learnt associations of spectrotemporal properties of sound with the psychological dimension of pitch height, as proposed in more recent pitch models by the authors that integrate spectral and periodicity based pitch models (McLachlan, 2009, 2011; McLachlan and Wilson, 2010; McLachlan et al., 2013). Thus, this study tested whether pitch-matching by people who have been trained with inharmonic gamelan percussion instruments was more reliable for these instruments than pitch-matching by people with similar levels of music training without exposure to gamelan instruments. A pilot study was first run to ensure that the gamelan musicians were better at recognizing gamelan instruments in the absence of a musical context.

## Methods

### Participants

Participants were recruited from gamelan ensembles at The University of Melbourne and Monash University, and compared with students from The Melbourne Conservatorium of Music (Table 1). There was no statistical difference in the years of western music training between the musician groups. All participants included in the gamelan musician groups had rehearsed with gamelan ensembles for more than 1 year. Information about the research was provided and written informed consent was obtained. No participant reported absolute pitch ability, abnormal hearing, or any serious neurological conditions.

**Table 1:** **Details of participants**.

	**Gamelan**	**Western**
No. Participants	10	34
No. Females (%)	4 (40)	16 (47)
Mean Years of Age (SD)	42.86 (18.25)	19.44 (3.02)
Mean Years of Training ^*^ (SD)	7.30 (9.59)	8.74 (5.72)

* Western music training only.

### Stimuli

All the instruments used in this study were recorded in a quiet room at a sound pressure level of 70 ± 2 dB(A) fast response at 1 m distance, and the recordings were edited to 500 ms length from the sound onset. The gamelan instruments belong to the central Javanese tradition (Kartomi, 1990). Four notes in the same octave of four types of gamelan instruments were used in a pilot study. These included small tuned gongs, metalophones, a xylophone, and a string zither. Since xylophone and string instruments are common in both gamelan and western music, the stimuli fell naturally into two equally sized groups comprising gamelan specific instruments (the gongs and metalophone) and common instruments (xylophone and string).

**Figure 1:**
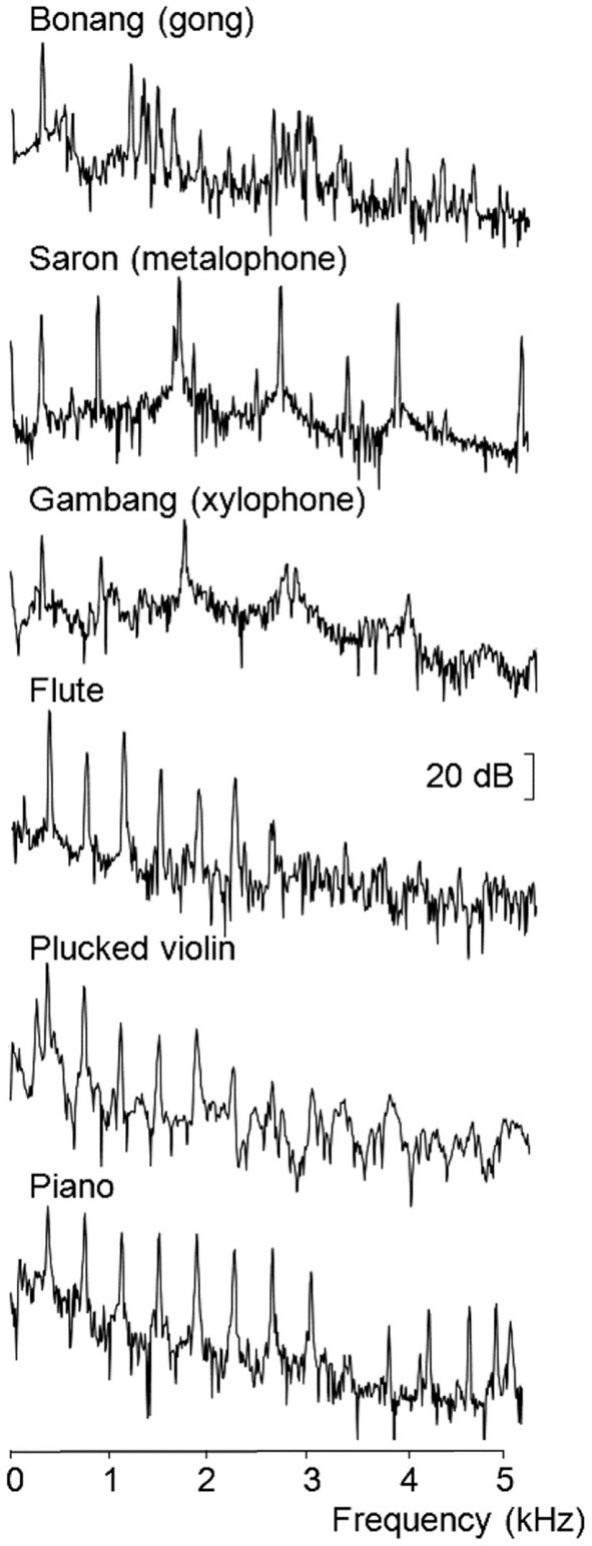
**FFT spectra recorded for the lowest frequency example of each instrument type (Hamming window over 4096 samples from sound onset at 44.1 kHz sampling rate)**.

The pitch-matching experiment used the same recordings of the gongs, metalophone, and xylophone. Acoustic spectra revealed that the lowest frequency partials of each note of the three gamelan instruments were all tuned within 2 Hz of each other, but the higher order inharmonic partials were at widely varying frequencies (Figure 1). Four notes of three western harmonic instruments with lowest frequency partials at similar frequencies to the gamelan instruments (D4, F4, A4, and B4) were also used in this experiment. These instruments included a flute, piano and plucked violin. The acoustic spectra shown in Figure 1 for each type of instrument used in the pitch-matching study reveals the distinctive inharmonic relationships of the overtones of the gamelan instruments compared to the harmonic overtones of the western instruments.

In the recognition pilot study participants were first trained using the recorded sounds with feedback (see procedure). We then increased the task difficulty by the addition of an onset noise mask. Onset noise masks of 150 ms duration full bandwidth white noise that was at least 3 dB greater in amplitude than any spectral component of the instrumental sound were applied to half of the stimuli from the stimulus onset.

### Procedure

The recognition task employed a forced-choice paradigm. Purpose built software displayed labeled images of the four instrument classes at equal distances from a central cross on a computer monitor. Participants clicked on the cross using a computer mouse to trigger an instrument sound and then clicked on the labeled image of their choice. In a training phase participants received visual feedback on the correct instrument after they had made their choice. Participants were trained until they achieved an overall accuracy of 90% correct over the previous 10 trials in the no mask condition before data collection commenced using the full set of masked and non-masked stimuli. The location of the instrument classes on the screen was randomized for each participant, as was the order of stimulus presentation. All participants completed the experiment.

**Figure 2:**
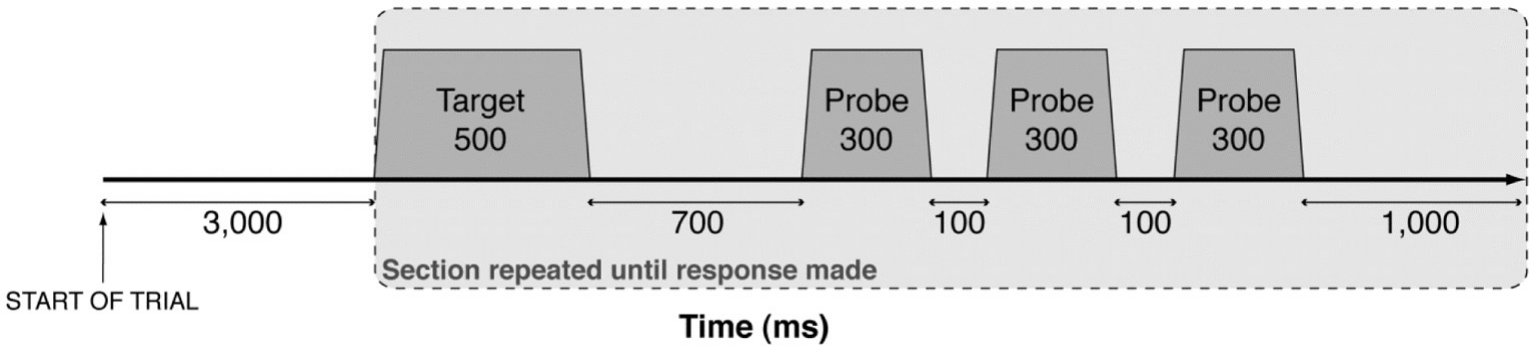
**A schematic representation of the presentation of auditory stimuli.** Each target stimulus and probe were synthesised with 30 ms linear onset and offset ramps and presented in a continuous sequence (gray shading) until participants matched the pitch of the probe to the target. Probes were synthesised in real-time at frequencies governed by participant movement of the computer mouse (axis not shown to scale).

The pitch-matching task was adapted from Moore and Glasberg (1985). Purpose-built computer software was used to present the stimuli and record task responses. Target stimuli were followed by a set of three pure tone probes, as shown in Figure 2. Bidirectional lateral movement of a computer mouse altered the pitch of the probe tones (right movement increased the pitch). The target stimulus and probe tones were repeated until the participant clicked the mouse to indicate when the probe tone matched the target, and the cycle was terminated. Pitch-matching accuracy was tested over 24 separate trials (6 instruments × 4 notes), pseudo-randomly ordered so that consecutive presentations of the same instrument or pitch were avoided. Trials were divided equally into two blocks each lasting approximately 10 min.

Participants completed training trials of the pitch-matching task using pure tone 1-pitch stimuli. Training continued up to a maximum of 10 trials with supervision until participants accurately matched a pure tone to within two semitones on three successive trials. Nine participants failed to reach this criterion and thus were excluded from experimental testing leaving the total of 44 participants described in Table 1. In the experimental trials, breaks were provided between blocks to minimize fatigue effects. All stimuli were presented to participants individually in an anechoic chamber at 70 ± 2 dB sound pressure level through two loudspeakers located on either side of a computer monitor (1 m in front and 0.5 m apart). Prior to the experimental trials, participants completed a questionnaire to collect information about their demographics, health, and musical background.

## Results

We first present the results of the recognition pilot study. In the absence of the onset mask the recognition rates of gamelan musicians were close to ceiling overall (94%). Western musicians performed at close to ceiling for the western instrument sounds (97%) but between 65–83% for the remaining instruments. In the presence of onset masks recognition rates generally fell to 50–70%, except for the western musician's performance for the gongs, which fell to chance levels (25%). A mixed within and between groups Analysis of Variance (ANOVA) of the rates of recognition with independent variables of instrumentation (two levels: gamelan specific and common) and musicianship (two levels: gamelan and western) revealed an interaction between instrumentation and musicianship [*F*_(1, 44)_ = 41.87, *p* < 0.01, *d* = 0.35], and main effects for both instrumentation [*F*_(1, 44)_ = 32.30, *p* < 0.001, *d* = 1.03], and musicianship [*F*_(1, 44)_ = 6.38, *p* < 0.01, *d* = 0.44]. Gamelan musicians were better for gamelan instruments (*M* = 51.50, *SD* = 4.98) than western musicians (*M* = 39.67, *SD* = 5.74) whereas there was no difference between the gamelan and western musicians for the western instruments (*M* = 65.21, *SD* = 6.54 and *M* = 61.09, *SD* = 6.82 respectively). These results confirm that the gamelan musicians were better at recognizing the gamelan specific instruments under the experimental conditions.

**Figure 3:**
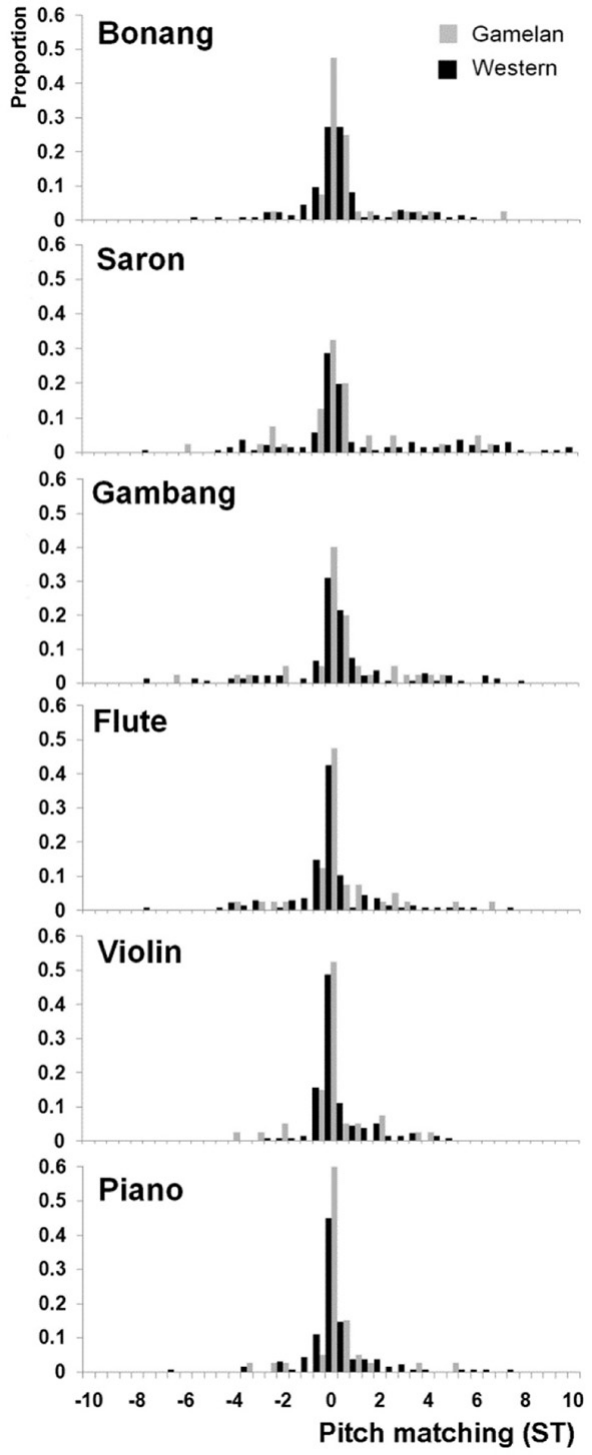
**Pitch-matching histograms for each instrument and musician group adjusted to the frequency of the first partial of each stimulus.** ST = semitones.

Inspection of the pitch matching distributions for each note of each instrument revealed that the median responses were always centered at the frequency of the lowest frequency partial. Therefore the data was analyzed by first subtracting the frequency of the lowest partial from each pitch matching response to generate distributions for each instrument type and musician group across all four pitches. This led to the normal distributions about 0 Hz shown in Figure 3. These data confirm that the pitch matching responses can be collapsed across the four notes used in the study by subtraction of the frequency of the first partial of each stimulus from the frequency of the tone matched by participant. Note that it was not possible to make octave errors for the stimuli with the pitch-matching apparatus.

**Figure 4:**
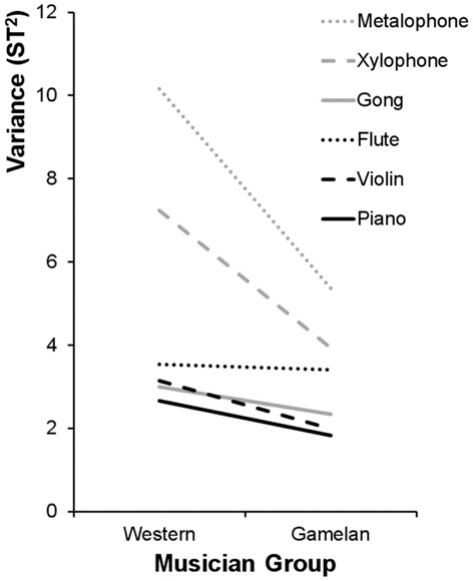
**Sample variance of the pitch matching distributions (cf. Figure 3) for each instrument type and each musician group.** ST = semitones.

Figure 4 shows that the variances of the pitch matching distributions for western musicians were larger than gamelan musicians for gamelan instruments but not western instruments. To evaluate these differences in variance, Brown–Forsythe Levene's tests were performed separately for the western and gamelan musicians across the six instruments. Separate Brown-Forsythe Levene's tests were also performed on the pitch matching distributions for the western and gamelan instruments for each musician group. The Brown-Forsythe Levene's test compares the absolute deviation of each pitch-matching trial with the median of the sample distribution, and so is robust to any systematic bias in the pitch matching distributions. The deviations from the group medians were analyzed using One-Way ANOVAs to enable comparisons between more than two distributions.

Results showed a significant difference in variance across the instruments for western musicians [*F*_(5, 810)_ = 10.55, *p* < 0.01] but not gamelan musicians [*F*_(5, 234)_ = 1.35, *p* = 0.25], and indicated that western musicians were poorer than gamelan musicians at pitch-matching the gamelan instruments [*F*_(2, 405)_ = 8.69, *p* < 0.01], but not western instruments [*F*_(2, 405)_ = 1.01, *p* = 0.37]. These results were confirmed by comparing the variance ratio to Hartley's Fmax critical value for the variances of the six instruments (Field, 2009).

To identify which instrument types differed significantly in variance for western musicians, *post hoc* Tamhane's T2 multiple comparisons were performed. Results showed that pitch-matching variance for the metalophone (*M* = 2.13, *SE* = 0.22) was higher than all other instruments except the xylophone (*M* = 1.55, *SE* = 0.19). The variance for the xylophone was significantly higher than the piano (*M* = 0.88, *SE* = 0.12) and violin (*M* = 0.81, *SE* = 0.14) but did not differ significantly from the gongs (*M* = 1.06, *SE* = 0.12) or flute (*M* = 1.06, *SE* = 0.13). There were no significant differences in variance between the gongs, flute, piano, and violin.

The pitch-matching distributions for the metalophone and xylophone showed greater spread than the other instruments. These are both keyed instruments with approximately equally spaced overtones, whereas the gongs had widely dispersed clusters of closely spaced overtones (Figure 1). Given the relatively low resolution of auditory nerve spectral responses (Figure 2) it is possible that the metalophone and xylophone were mistaken for harmonic spectra by western musicians, leading to higher pitch-matching variance. In contrast, both groups of musicians performed better for the gongs, possibly as a result of focusing on the frequency region of the relatively isolated, lowest frequency partial.

As shown in Figure 4, the pitch-matching variance for all instruments appears high in light of the high level of music training of the participants. However, for the western instruments, 74% of pitch-matching responses were within one semitone, which is consistent with pitch just noticeable difference limens reported by Moore (1973) of around 1% (or approximately 0.2 semitones) for synthesized tones that contain less timbral complexity.

## Discussion

This study showed that western musicians with gamelan training were better at recognizing gamelan instruments, and more consistent at matching their pitch at the lowest frequency partial than musicians with a similar level of training, but no exposure to gamelan. No differences in performance were found between musician groups for western instruments, indicating that the better performance by the gamelan musicians for gamelan instruments was not a result of superior pitch matching ability *per se*. Note, however, that this was based on a relatively small sample of 10 gamelan musicians. Given that gamelan musicians were also better at recognizing gamelan instruments, these findings suggests that gamelan musicians were able to use recognition templates for these inharmonic instruments to rapidly prime pitch processing for inharmonic timbres.

McLachlan (2009, 2011) proposed that template matching by recognition mechanisms might precede fine pitch processing by periodicity mechanisms, thereby allowing periodicity to be integrated at just one frequency that is primed by recognition of the stimulus onset spectrum. The initial pitch model was also expanded into a more general account of auditory processing (the Object-Attribute model, McLachlan and Wilson, 2010) that proposed that recognition of the sound type (e.g., a pure or complex tone) involves correlation of sequential spectral slices of stimulus driven neural activity with long-term memory templates. More recently this model has been supported by behavioral data that shows better pitch matching by musicians for familiar music chords, and improved pitch matching associated with learning to pitch stimuli comprising individual pure tones and chords of pure tones (McLachlan et al., 2013). Furthermore, pitch matching distributions for chords were always most accurate for the highest pitch, suggesting that templates initially prime just the highest pitch of a chord (McLachlan et al., 2013). Given that gamelan instruments are tuned by the frequency of their first partial, gamelan players have likely developed a local network for each instrument to prime pitch at the lowest frequency partial. This would allow periodicity information from just this partial to contribute to the fine pitch resolution ability displayed by the gamelan musicians. In contrast, western musicians may have attempted to use harmonic templates to prime the pitch of gamelan instruments, since these templates would be the closest match they have to the instrument timbres, leading to high variability in pitch matching responses.

Neither periodicity pitch models (Licklider, 1951; Meddis and Hewitt, 1991; Cariani and Delgutte, 1996; Meddis and O'Mard, 2006), nor harmonic template matching models (Goldstein, 1973; Terhardt, 1974; Parncutt, 1989) can account for the gamelan musician performance observed in this study. Periodicity models integrate information across all auditory filter channels, and so conflicting pitch information from inharmonic partials would be included in pitch representations. Harmonic template matching models include both synthetic (or virtual) pitch processing by harmonic template fitting, and analytic pitch percepts associated with the salience of individual partials. In these models experimentally derived weights are used to account for the dominance region for virtual pitch (Ritsma, 1962; Terhardt et al., 1982b), and a further weighting is applied to alter the salience of synthetic pitches relative to analytic pitches.

Terhardt et al. (1982a) and Parncutt (1989) attempted to use harmonic template models to explain pitch perception of inharmonic bell sounds by adjusting the model weights to fit limited experimental data. To account for the finding that gamelan musicians made accurate pitch matches to the lowest frequency partials of the inharmonic gamelan instruments, all harmonic template model weights would need to be set to zero apart from the analytical pitch salience weight applied to the lowest frequency partial. Furthermore, these weights would need to be altered for inharmonic stimuli and then restored for harmonic stimuli, unless musicians lose their ability to hear virtual pitch for harmonic sounds when they learn gamelan music. In other words, the stimuli would first need to be recognized as being inharmonic so that pitch weightings could be altered accordingly. It seems more parsimonious to propose that the stimulus is compared to a stored representation or template that rapidly primes only the lowest frequency partial, as proposed in McLachlan (2009), regardless of whether that template contains harmonic or inharmonic partials. This is consistent with experimental data and models described by McLachlan and colleagues in which recognition mechanisms rapidly associate spectral information with a low resolution pitch height estimate according to learnt musical relationships. This initial pitch height estimate is then used to prime periodicity pitch processing mechanisms by inhibiting periodicity responses at other frequencies (McLachlan and Wilson, 2010; McLachlan, 2011; McLachlan et al., 2013).

Culturally defined differences in musical scale processing have been observed in Western and Indian musicians (Castellano et al., 1984; Bigand et al., 2005), and implicit learning of pitch intervals of pure tones has been described (Loui et al., 2010). However previous research on pitch processing has not considered the possibility that pitch associations may also be learnt for stimulus spectrum, leading to the assumption that pitch is based only on harmonically related partials. The present findings suggest that pitch processing is more adaptable than earlier models suggest, and instead support the idea that the association of pitch height with stimulus spectra may be subserved by spectral recognition mechanisms. Since these associations are learnt by the deliberate pairing of a pitch with a sound spectrum, it is conceivable that the music environment can influence the ability to perceive pitch within a specific music tradition.

### Conflict of interest statement

The authors declare that the research was conducted in the absence of any commercial or financial relationships that could be construed as a potential conflict of interest.
